# What is the lowest change in cardiac output that transthoracic echocardiography can detect?

**DOI:** 10.1186/s13054-019-2413-x

**Published:** 2019-04-11

**Authors:** Mathieu Jozwiak, Pablo Mercado, Jean-Louis Teboul, Anouar Benmalek, Julia Gimenez, François Dépret, Christian Richard, Xavier Monnet

**Affiliations:** 1 Hôpitaux Universitaires Paris-Sud, Hôpital de Bicêtre, APHP, Service de Médecine Intensive-Réanimation et de Surveillance Continue Médicale, 78, rue du Général Leclerc, F-94270 Le Kremlin-Bicêtre, France; 2 Inserm UMR S_999, Univ Paris-Sud, 78, rue du Général Leclerc, F-94270 Le Kremlin-Bicêtre, France; 3 Faculté de Pharmacie, Université Paris-Sud, 5 rue Jean-Baptiste Clément, F-92290 Châtenay-Malabry, France

**Keywords:** Fluid challenge, Fluid responsiveness, Intensive care unit, Ultrasound, Velocity-time integral

## Abstract

**Background:**

In critically ill patients, changes in the velocity-time integral (VTI) of the left ventricular outflow tract, measured by transthoracic echocardiography (TTE), are often used to non-invasively assess the response to fluid administration or for performing tests assessing fluid responsiveness. However, the precision of TTE measurements has not yet been investigated in such patients. First, we aimed at assessing how many measurements should be averaged within one TTE examination to reach a sufficient precision for various variables. Second, we aimed at identifying the least significant change (LSC) of these variables between successive TTE examinations.

**Methods:**

We prospectively included 100 haemodynamically stable patients in whom TTE examination was planned. Three TTE examinations were performed, the first and the third by one operator and the second by another one. We calculated the precision and LSC (1) within one examination depending on the number of averaged measurements and (2) between measurements performed in two successive examinations.

**Results:**

In patients in sinus rhythm, averaging three measurements within an examination was enough for obtaining an acceptable precision (interquartile range highest value < 10%) for VTI. In patients with atrial fibrillation, averaging five measurements was necessary. The precision of some other common TTE variables depending on the number of measurements is provided. Between two successive examinations performed by the same operator, the LSC was 11 [5–18]% for VTI. If two operators performed the examinations, the LSC for VTI significantly increased to 14 [8–26]%. The LSC between two examinations for other TTE variables is also provided.

**Conclusions:**

Averaging three measurements within one TTE examination is enough for obtaining precise measurements for VTI in patients in sinus rhythm but not in patients with atrial fibrillation. Between two TTE examinations performed by the same operator, the LSC of VTI is compatible with the assessment of the effects of a 500-mL fluid infusion but is not precise enough for assessing the effects of some tests predicting preload responsiveness.

**Electronic supplementary material:**

The online version of this article (10.1186/s13054-019-2413-x) contains supplementary material, which is available to authorized users.

## Background

Today, transthoracic echocardiography (TTE) is widely used for evaluating the haemodynamic condition in the intensive care unit (ICU) and the operating theatre [1–4]. In particular, in critically ill patients, TTE is daily used to track changes in cardiac output, which are assessed from relative changes in the velocity-time integral (VTI) of the left ventricular outflow tract. Especially, TTE is used for assessing the effects on cardiac output of some therapeutic interventions (fluid administration, inotrope infusion) [5–10] or for performing tests assessing preload responsiveness like the end-expiratory and inspiratory occlusion tests [11, 12], passive leg raising [7, 9, 13–15] or a mini-fluid challenge [8].

Nevertheless, such changes in cardiac output are sometimes of small amplitude. In particular, the diagnostic threshold of changes induced by tests of fluid responsiveness is relatively small, ranging from 5% [11, 12] to 10% [8, 14]. Thus, the precision of TTE measurements is of tremendous importance. While accuracy indicates the bias between measurements and the true value they estimate, precision indicates how measurements are close to each other [16]. However, precision has not been fully investigated in critically ill patients, except through inter-observer and intra-observer variability [7, 8, 11, 13, 17]. Nevertheless, such analyses do not allow one to answer the twofold question raised by the issue of precision.

The first one is to know how many measurements should be averaged within one TTE examination by the same operator in order to obtain measurements of a sufficient precision level, which is usually fixed at 10% [16]. Then, the first goal of this study was to assess the precision of the VTI within one examination by the same operator, without removing the probe from the patient (intra-examination analysis). The second question is to know the minimal change between two TTE examinations that can be considered as significant. The second goal of this study was thus to assess the least significant change (LSC) of the VTI between two examinations, performed either by the same or by different operators (inter-examination analysis). Since other TTE variables might have a potential clinical interest at the bedside in ICU or in the cardiology ward, we extended our study to the other most common TTE variables.

## Methods

This study was conducted in a 25-bed medical ICU and approved by the Institutional Review Board of our institution (Comité pour la protection des personnes Ile de France VII, number IDRCB 2016-A00939-42). All patients or next of kin were informed about the study and consented to participate.

### Patients

We included consecutive patients with haemodynamic stability (no change in the norepinephrine dose and changes in systolic arterial pressure < 10% within 5 min before the inclusion) for whom a TTE examination was planned. The dosage of norepinephrine and sedatives was unchanged during the study period. The exclusion criterion was poor echogenicity, defined as the inability to correctly align the Doppler beam to obtain reliable Doppler measurements and/or to correctly delineate the endocardium for measuring the left ventricular ejection fraction (LVEF).

### Echocardiographic measurements

Three successive TTE examinations were performed by two operators, the first and the third by one operator and the second by the other. Within each examination, measurements were performed without removing the probe from the thorax.

From apical five- and four-chamber views, we measured the VTI, the LVEF calculated by the modified Simpson’s rule, the early (*E*) and atrial (*A*) peak velocities of the transmitral flow with pulsed Doppler, the early diastolic (*e*’) and systolic (*s*’) peak velocities of the lateral mitral annulus and the systolic peak (*S*) velocity of the tricuspid annulus with tissue Doppler imaging, the tricuspid annular plane systolic excursion (TAPSE) in the M-mode and the left and right ventricular end-diastolic area (LVEDA and RVEDA). From these variables, we calculated the *E*/*A*, *E*/*e*’ and RVEDA/LVEDA ratios. All contours were hand-drawn.

All echocardiographic measurements were performed with a Philips CX 50 (Philips Healthcare, DA Best, The Netherlands) by four different board-certified operators (MJ, PM, JG, FD) at end-expiration and according to current recommendations [18–20].

### Assessment of precision

#### Intra-examination analysis

**Fig. 1:**
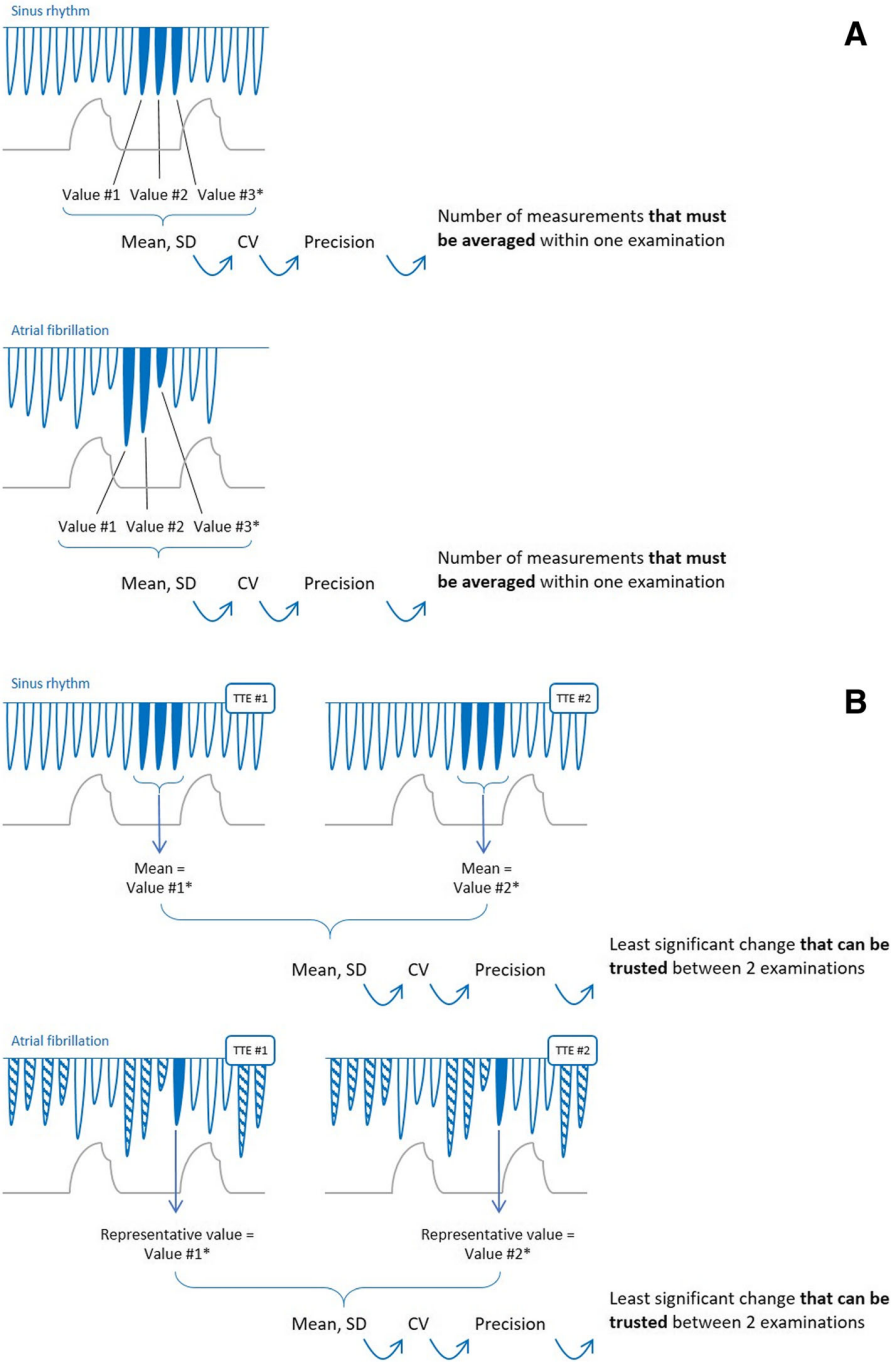
Method for assessing intra-examination precision (**a**) and inter-examination least significant change (**b**). CV coefficient of variation, SD standard deviation. Asterisk indicates that for the sake of simplicity, the figure is presented as if all end-expiratory cycles were consecutive. If we could not record enough cycles during the end-expiratory period of one cycle, the values recorded during the end-expiration in several cycles were used

This analysis was performed for determining the number of measurements to average within one TTE examination (first goal of the study). The principle of this analysis is that, in an experimental population, a series of measurements is obtained. The value of standard deviation (SD) of these measurements is calculated for each variable (Fig. 1a). From this SD, which comes from the *real* measurements performed, one infers from formulas the precision and the LSC for each variable. They are general characteristics to which one should refer for any measurement. The precision improves and the LSC decreases along with the number of measurements that are averaged. Since they are obtained from formulas which take the repetition of measurements into account, one can calculate them for any theoretical number of measurements averaged, even though the experimental sample that allowed the calculation of SD was made of three values only (Fig. 1a).

In practice, at a first step, the SD was obtained from the three consecutive end-expiratory measurements that had been performed, for patients in sinus rhythm as for patients with atrial fibrillation. The corresponding coefficient of variation (CV) was calculated as CV = SD/mean of the three measurements [21] (Fig. 1a).

At a second step, from the CV obtained in the experimental population, we inferred the coefficient of error (CE) for each variable as CE = CV/√*n*, where *n* is the number of replicates that the TTE examination performer would choose to average *in theory* within one TTE examination. The precision was calculated as being 2 CE [21–23]. The lower the value of the calculated precision, the closer the measurements to each other, and the more precise the technique [16]. It is usually considered that a precision level < 10% is desirable [16]. The intra-examination LSC was calculated using the following equation: LSC = CE × 1.96 × √2 [21, 22, 24]. This corresponds to the minimal change observed *during an examination* that can be considered as real and not related to the variability of the measurement.

In addition, the intra-examination intra-observer variability, which is another way to estimate reproducibility of measurements, was expressed as the mean percentage error. It was calculated as the difference between two consecutive end-expiratory measurements within a TTE examination divided by the mean of both values.

#### Inter-examination analysis

This analysis was performed for determining the minimal change that can be regarded as significant *between two successive TTE examinations* (second goal of the study). The principle was the same as for the intra-examination analysis except that the SD was calculated not for the measurements performed within one examination, but for the average of the measurements obtained in each examination.

In practice, at a first step, the SD was obtained from the results of the two examinations. This result was the average of three consecutive end-expiratory measurements performed within one examination in patients in sinus rhythm [18, 19, 25–27] or a single end-expiratory measurement visually estimated as the average of ten consecutive end-expiratory ones performed within one examination in patients with atrial fibrillation [28, 29]. The corresponding coefficient of variation (CV) was calculated as CV = SD/mean of the two measurements, each being performed in one TTE [21] (Fig. 1b).

At a second step, from this CV, we calculated the inter-examination CE, precision and LSC of variables of interest by using the formulas cited above. All these calculations were performed for the same as well as for different operators.

In addition, the inter-examination intra-observer variability was calculated as the difference between two end-expiratory measurements obtained in two consecutive TTE examinations performed by the same operator divided by the mean of both values. The inter-examination inter-observer variability was calculated as the difference between two end-expiratory measurements obtained in two consecutive TTE examinations performed by two different operators divided by the mean of both values.

### Statistical analysis

Normality of data was assessed by a Kolmogorov-Smirnov test. Within groups, variables were compared by a paired Student *t* test or a Wilcoxon signed-rank test. Between groups, a two-tailed Student *t* test, a Wilcoxon Mann-Whitney *U* test or a Fisher’s exact test was used. We planned a priori subgroup analyses between patients in sinus rhythm and with atrial fibrillation, and between patients with and without invasive mechanical ventilation.

We estimated that including 100 patients should allow us to obtain a reliable value of intra- and inter-examination SD for all the TTE variables of interest. Statistical analysis was performed with MedCalc 11.6.0 software (MedCalc, Mariakerke, Belgium). A *p* value < 0.05 was considered statistically significant.

## Results

### Study population

One hundred and ten patients fulfilled the inclusion criteria. Among the 110 included patients, 18 had atrial fibrillation, 59 were under invasive mechanical ventilation, 31 were sedated, 37 received norepinephrine and 3 received dobutamine. Ten patients were excluded due to poor echogenicity. No patient received neuromuscular blockers nor had a pacemaker. The proportion of patients with invasive mechanical ventilation was 39% among patients with atrial fibrillation and 57% among patients in sinus rhythm (*p* = 0.17). The proportion of patients with atrial fibrillation was 12% in patients with invasive mechanical ventilation and 22% in patients without (*p* = 0.17). The ICU mortality was 17%. The other baseline characteristics are summarised in Additional file 1.

### Indication of echocardiographic measurements

Among the 100 analysed patients, the indication for TTE examination was the assessment of LVEF in 65 patients, the estimation of the left ventricular filling pressure in 23 patients, the suspicion of *acute cor pulmonale* in 5 patients, the search of the cause of tachycardia in 4 patients, the suspicion of endocarditis in 2 patients and the suspicion of *patent foramen ovale* in 1 patient. The mean time elapsed between the first and the third TTE examinations was 19 ± 11 min. Haemodynamic variables were similar between the first and the third TTE examinations (Additional file 2).

### Measurements within a TTE examination in patients in sinus rhythm

**Fig. 2:**
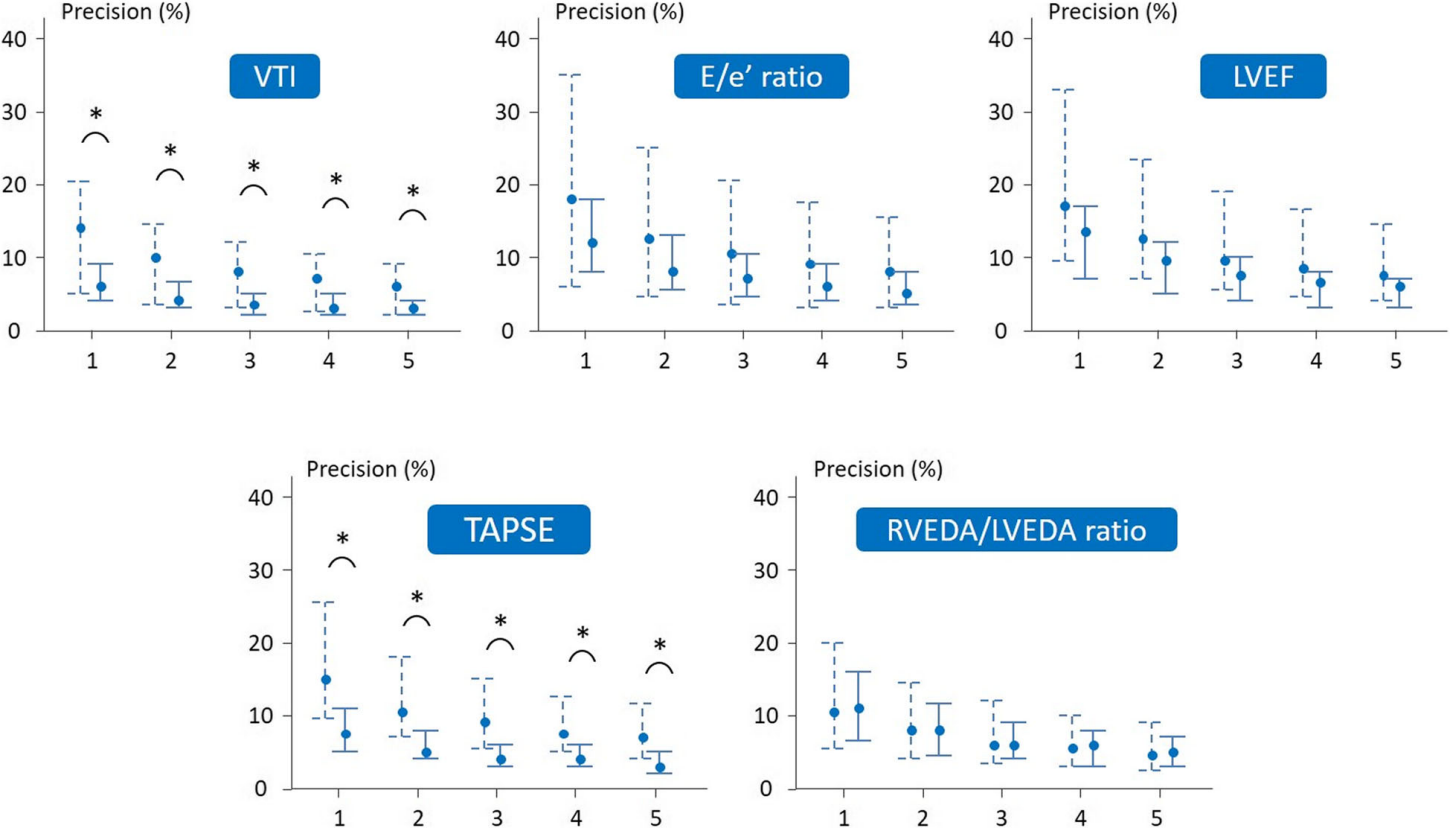
Intra-examination precision according to the number of measurements averaged within one transthoracic echocardiography examination. Data are expressed as median and interquartile ranges. *p < 0.05 sinus rhythm vs. atrial fibrillation. Solid lines indicate patients in sinus rhythm (*n* = 84). Dashed lines indicate patients with atrial fibrillation (*n* = 16). *E*/*e*’ ratio, ratio of the early peak velocity of transmitral flow over the early diastolic peak velocity of the lateral mitral annulus; LVEF, left ventricular ejection fraction; RVEDA/LVEDA ratio, ratio of the end-diastolic right over left ventricular areas; TAPSE, tricuspid annular plane systolic excursion; VTI, velocity-time integral

In patients in sinus rhythm, if the echocardiographer chose to measure only one VTI, the intra-examination precision would be 6 [4–9]%. It would decrease to 4 [2–5]% if the echocardiographer would average three measurements (Table 1, Fig. 2). In this case, the median value of precision would be < 10% for all the studied variables. The highest value of the interquartile range would be < 10% for all variables except LVEF and *E*/*e*’ ratio (Table 1, Fig. 2). The LSC for measurements within a TTE examination in patients in sinus rhythm are provided in Additional file 3 and the intra-observer variability in Additional file 4.

**Table 1:** Intra-examination precision of transthoracic echocardiography measurements in sinus rhythm

TTE parameters	One measurement	Two measurements	Three measurements	Four measurements	Five measurements
LV parameters					
*E* wave	7 [4–11]%	5 [3–8]%	4 [2–6]%	4 [2–6]%	3 [2–5]%
*A* wave	8 [4–11]%	6 [3–8]%	5 [2–6]%	4 [2–5]%	6 [3–8]%
*e*’ wave	9 [5–14]%	6 [3–10]%	5 [3–8]%	5 [2–7]%	4 [2–6]%
*E*/*A* ratio	10 [5–14]%	7 [4–10]%	6 [3–8]%	5 [3–7]%	4 [2–6]%
*E*/*e*’ ratio	12 [8–18]%	8 [6–13]%	7 [5–11]%	6 [4–9]%	5 [4–8]%
*s*’ wave	9 [6–13]%	6 [4–9]%	5 [3–8]%	4 [3–7]%	4 [3–6]%
VTI	6 [4–9]%	4 [3–7]%	4 [2–5]%	3 [2–5]%	3 [2–4]%
LVEF	14 [7–17]%	10 [5–12]%	8 [4–10]%	7 [3–8]%	6 [3–7]%
RV parameters					
TAPSE	7 [5–11]%	5 [4–8]%	4 [3–6]%	4 [3–6]%	3 [2–5]%
*S* wave	8 [4–12]%	6 [3–8]%	5 [2–7]%	4 [2–6]%	3 [2–5]%
LV and RV dimensions					
LVEDA	7 [5–11]%	5 [3–8]%	4 [3–6]%	4 [2–5]%	3 [2–5]%
RVEDA	9 [6–14]%	7 [4–10]%	5 [3–8]%	5 [3–7]%	4 [3–6]%
RVEDA/LVEDA	11 [7–16]%	8 [5–12]%	6 [4–9]%	6 [3–8]%	5 [3–7]%

*n* = 84, data are summarised as median [interquartile range]

*LV* left ventricular, *RV* right ventricular, *TTE* transthoracic echocardiography, *E* early peak velocity of transmitral flow with pulsed Doppler, *A* atrial peak velocity of transmitral flow with pulsed Doppler, *e’* early diastolic peak velocity of the lateral mitral annulus with tissue Doppler imaging, *s’* systolic peak velocity of the lateral mitral annulus with tissue Doppler imaging, *VTI* velocity-time integral of the left ventricular outflow tract, *LVEF* left ventricular ejection fraction, *TAPSE* tricuspid annular plane systolic excursion, *S* systolic peak velocity of the tricuspid annulus with tissue Doppler imaging, *LVEDA* left ventricular end-diastolic area, *RVEDA* right ventricular end-diastolic area

### Measurements within a TTE examination in patients with atrial fibrillation

In patients with atrial fibrillation, the intra-examination precision for VTI, *e*’ wave, TAPSE and *S* wave was worse than in patients in sinus rhythm, whatever the number of measurements that the echocardiographer would choose to average (Additional file 5, Fig. 2). If five measurements would be averaged, the median value of precision would be < 10% for all variables. In this case, the highest value of the interquartile range would be < 10% for all variables except LVEF, *e*’ wave, TAPSE and *E*/*e*’ ratio. The LSC for measurements within a TTE examination in patients with atrial fibrillation are provided in Additional file 3 and the intra-observer variability in Additional file 4. Intra-examination precision did not depend on the operator whatever the cardiac rhythm (data not shown).

### Measurements within a TTE examination in patients with invasive ventilation

In patients with invasive ventilation, except for *A* and *e*’ waves and the *E*/*A* ratio, the precision was similar to that observed in patients without invasive ventilation, whatever the number of measurements that the echocardiographer would choose to average (Additional file 6). The LSC for measurements within a TTE examination in patients with invasive ventilation are provided in Additional file 7 and the intra-observer variability in Additional file 4.

### Measurements between two TTE examinations performed by the same operator

When TTE examinations were performed by the same operator, the inter-examination LSC was 11 [5–18]% for VTI, 8 [4–15]% for the LVEF, 24 [9–41]% for the *E*/*e*’ ratio, 14 [6–27]% for the TAPSE and 17 [9–30]% for the RVEDA/LVEDA ratio (Table 2). The inter-examination LSC for the other TTE variable measurements are summarised in Table 2 and the precision and intra-observer variability in Additional file 8.

**Table 2:** Least significant change of transthoracic echocardiography measurements between two examinations

TTE parameters	Same operator	Different operator
LV parameters		
*E* wave	8 [4–17]%	11 [5–19]%*
*A* wave^£^	9 [3–18]%	13 [6–23]%*
*e*’ wave	16 [7–34]%	19 [9–38]%
*E*/*A* ratio^£^	11 [6–19]%	11 [5–25]%
*E*/*e*’ ratio	24 [9–41]%	21 [10–39]%
*s*’ wave	14 [7–23]%	18 [8–27]%
VTI	11 [5–18]%	14 [8–26]%*
LVEF	8 [4–15]%	8 [3–19]%
RV parameters		
TAPSE	14 [6–27]%	17 [7–33]%
*S* wave	12 [6–24]%	15 [7–31]%*
LV and RV dimensions		
LVEDA	12 [7–21]%	15 [7–26]%
RVEDA	15 [6–31]%	17 [10–33]%
RVEDA/LVEDA ratio	17 [9–30]%	17 [7–35]%

*n* = 100, data are summarised as median [interquartile range]

*LV* left ventricular, *RV* right ventricular, *TTE* transthoracic echocardiography, *E* early peak velocity of transmitral flow with pulsed Doppler, *A* atrial peak velocity of transmitral flow with pulsed Doppler, *e’* early diastolic peak velocity of the lateral mitral annulus with tissue Doppler imaging, *s’* systolic peak velocity of the lateral mitral annulus with tissue Doppler imaging, *VTI* velocity-time integral of the left ventricular outflow tract, *LVEF* left ventricular ejection fraction, *TAPSE* tricuspid annular plane systolic excursion, *S* systolic peak velocity of the tricuspid annulus with tissue Doppler imaging, *LVEDA* left ventricular end-diastolic area, *RVEDA* right ventricular end-diastolic area

*p < 0.05 different vs. same operator

^£^Concerning the *A* wave and the *E*/*A* ratio, *n* = 84

**Fig. 3:**
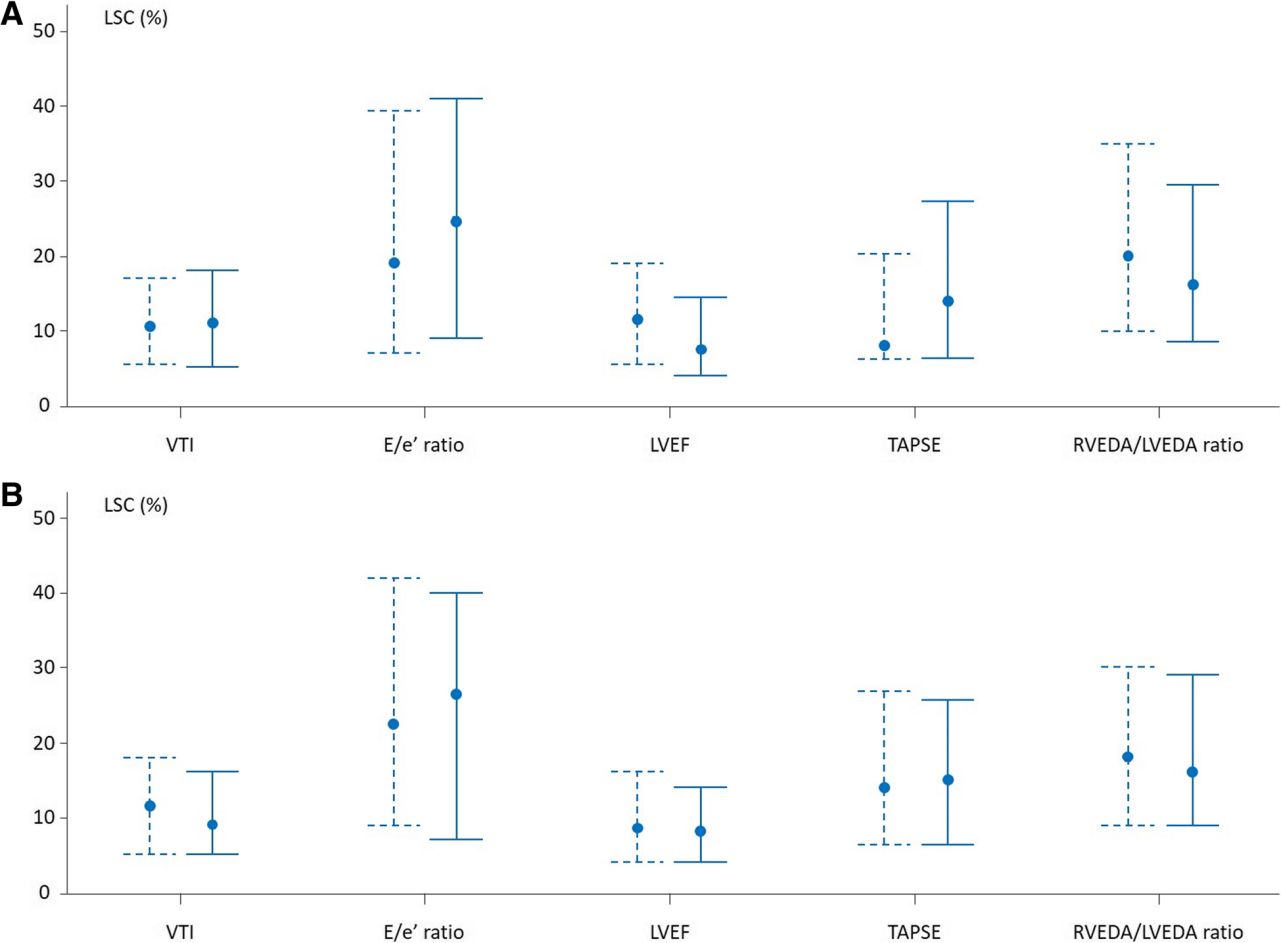
Inter-examination least significant change (LSC) between two transthoracic echocardiography examinations performed by the same operator. Data are expressed as median and interquartile ranges. **a** Solid lines indicate patients in sinus rhythm (*n* = 84). Dashed lines indicate patients with atrial fibrillation (*n* = 16). **b** Solid lines indicate patients without invasive mechanical ventilation (*n* = 46). Dashed lines indicate patients with invasive mechanical ventilation (*n* = 54). *E*/*e*’ ratio, ratio of the early peak velocity of transmitral flow over the early diastolic peak velocity of the lateral mitral annulus; LVEF, left ventricular ejection fraction; RVEDA/LVEDA ratio, ratio of the end-diastolic right over left ventricular areas; TAPSE, tricuspid annular plane systolic excursion; VTI, velocity-time integral

Whatever the TTE variables, the inter-examination LSC was similar between patients with sinus rhythm and atrial fibrillation (Fig. 3a, Additional file 9) and with and without invasive ventilation (Fig. 3b, Additional file 10).

### Measurements between two TTE examinations performed by two operators

When TTE examinations were performed by two operators, the inter-examination LSC was significantly higher for *E*, *A* and *S* waves and VTI than when TTE examinations were performed by the same operator (Table 2). The inter-examination LSC for the other TTE variable measurements are summarised in Table 2 and the precision and inter-observer variability in Additional file 8.

The inter-examination precision and LSC were similar between patients with sinus rhythm and atrial fibrillation for all TTE variables except the *s*’ wave. They were similar regardless which operator performed examinations (data not shown).

## Discussion

For performing TTE in critically ill patients, one should know how many measurements to average within one examination and how clinicians could be confident regarding the changes they measured between two examinations. We observed that averaging three measurements within one TTE examination in patients in sinus rhythm was enough for obtaining a precision < 10%, which is usually considered as desirable [16], for the majority of the common TTE variables. In patients with atrial fibrillation, averaging five measurements was necessary. Between two TTE examinations performed at different times by the same operator, the LSC was 11% for VTI. It ranged from 8% for LVEF to 24% for the *E*/*e*’ ratio. It significantly increased for VTI to 14% if two operators performed examinations, but did not change for most other variables.

The variability of echocardiographic measurements might be explained by three factors. The first is the variation in the position of the ultrasound beam with respect to cardiac structures. This factor of variability is important when the probe position has been changed, like between different examinations. The second resides in the variability, on a given image, in the placement of markers (contours, callipers) on the Doppler profile, M-mode or 2D images. This factor is influenced by the quality of the images and signals, which is often decreased in ICU patients. The third is the intrinsic variability of the variables (cardiac arrhythmias, irregular ventilation). Our study aimed at investigating these factors, especially by looking at the intra- and inter-examination precision of TTE variables. For this purpose, though the reproducibility of TTE is usually investigated through inter-observer and intra-observer variability [7, 8, 11, 13, 17], we chose a method based on calculation of measurements SD, allowing the calculation of CV [30]. Compared to inter- and intra-variability calculation, it allows the assessment of the effect of repeating measurements [30].

The LSC is the smallest change that can be considered as significant and not related to the imprecision of the method or the variability of the parameter. The intra-examination LSC is important for interpreting the relative changes in ultrasound indices when the sonographer performs measurements without removing the probe from the chest wall. This happens for instance when assessing the respiratory variation of VTI, for detecting tamponade or testing fluid responsiveness, or when performing respiratory occlusion tests [11, 12] and recruitment manoeuvres [31]. Our study shows that changes in VTI smaller than 5% cannot be considered as relevant. For the end-expiratory occlusion test, clinicians may also consider combining end-expiratory and end-inspiratory occlusion tests in order to increase the amplitude of the induced changes in VTI [11] much over the LSC threshold.

The inter-examination LSC is even more interesting to consider because it regards the several instances when one assesses changes in echocardiography variables during two different examinations, by different operators or by the same operator, but after replacing the probe on the thorax. In particular, a positive response to fluid is often defined by an increase in VTI ≥ 15% [5–10]. This threshold seems reasonable in most patients when measurements are performed by the same operator (VTI LSC 11 [5–18]%), but obviously to small if performed by different ones.

This is also the case for the passive leg raising test [7, 9, 13–15] or the 100-mL fluid challenge [8], the positivity of which is defined by an increase in VTI ≥ 10%, even though this threshold is very close to the VTI LSC for examinations performed by the same operator. By contrast, the VTI LSC is larger than the diagnostic threshold of VTI changes found for fluid challenges smaller than 100 mL [32], or for the end-expiratory occlusion test [11, 31, 33]. In such instances, changes in VTI must be assessed by the same operator, without moving the probe during the whole duration of the test.

Regarding the number of measurements to average within one examination, we found that, within one examination in patients in sinus rhythm, the precision was acceptable for almost all variables if measurements were averaged over three cardiac cycles, the median and the highest value of the interquartile range being < 10% [16, 22]. This agrees with the most recent cardiology recommendations [18, 19, 25–27]. Interestingly, the variables with the worst precision were LVEF and the *E*/*e*’ ratio, which precision itself depends on the precision of the measurements used for calculating them. Importantly, in order to minimise the intervention of respiratory variations in measurements during experimental data acquisition, we performed measurements at end-expiration. In this regard, the preload status of the patients should not significantly interfere with our results. The values of precision and LSC we provide are thus valid only in a similar condition.

In patients with atrial fibrillation, averaging five measurements was necessary for reaching a median precision < 10% for all the variables, though the highest value of the interquartile range was higher for some of them. Current recommendations are to average measurements over a minimum of five cycles [18] or over ten cycles [20] in case of atrial fibrillation. This is not so discrepant with our findings, because recommendations have been established for cardiology patients, which heart rate is often less, and thus, measurement variability is higher than in critically ill patients.

A first limitation of this study was that TTE examinations were performed by four operators, which might have accounted for part of the variability. Nevertheless, whatever the TTE variables, the intra- and inter-examination precision was similar for all operators. Second, we did not investigate to which extent echogenicity is a factor that may influence precision. Third, for feasibility reasons, we did not investigate all the variables that can be measured by TTE. Fourth, only a limited number of patients with atrial fibrillation were included and further studies are required to confirm our results in such patients. Nevertheless, our results agree with the most recent recommendations.

## Conclusions

In critically ill patients in sinus rhythm, averaging three measurements within one TTE examination is enough for obtaining precise measurements for the majority of the common TTE variables. In patients with atrial fibrillation, averaging five measurements is necessary. Between two TTE examinations performed at different times by the same operator, the least change of VTI is compatible with the assessment of the effects of a 500-mL fluid infusion, but is not precise enough for assessing the effects of some tests that have been developed for predicting fluid responsiveness.

## Additional files

Additional file 1: **Table S1.** Patients’ characteristics at baseline. (DOCX 33 kb)

Additional file 2: **Table S2.** Haemodynamic parameters during the first and the third transthoracic echocardiography examinations. (DOCX 25 kb)

Additional file 3: **Table S3.** Intra-examination least significant change of transthoracic echocardiography measurements according to cardiac rhythm. (DOCX 28 kb)

Additional file 4: **Table S4.** Intra-examination intra-observer variability of transthoracic echocardiography measurements according to cardiac rhythm and mechanical ventilation. (DOCX 26 kb)

Additional file 5: **Table S5.** Intra-examination precision of transthoracic echocardiography measurements in atrial fibrillation. (DOCX 26 kb)

Additional file 6: Table S6. Intra-examination precision of transthoracic echocardiography measurements according to mechanical ventilation. (DOCX 28 kb)

Additional file 7: **Table S7.** Intra-examination least significant change of transthoracic echocardiography measurements according to mechanical ventilation. (DOCX 30 kb)

Additional file 8: **Table S8.** Precision, intra- and inter-observer variability of transthoracic echocardiography measurements between two examinations. (DOCX 31 kb)

Additional file 9: **Table S9.** Variability of transthoracic echocardiography measurements between two examinations performed by the same operator according to cardiac rhythm. (DOCX 27 kb)

Additional file 10: **Table S10.** Variability of transthoracic echocardiography measurements between two examinations performed by the same operator according to mechanical ventilation. (DOCX 27 kb)

## Abbreviations

*A*: Atrial peak velocity of the transmitral flow with pulsed Doppler
CE: Coefficient of error
CV: Coefficient of variation
*E*: Early peak velocity of the transmitral flow with pulsed Doppler
*e*’: Early diastolic peak velocity of the lateral mitral annulus with tissue Doppler imaging
ICU: Intensive care unit
LSC: Least significant change
LVEDA: Left ventricular end-diastolic area
LVEF: Left ventricular ejection fraction
RVEDA: Right ventricular end-diastolic area
*S*: Systolic peak velocity of the tricuspid annulus with tissue Doppler imaging
*s*’: Systolic peak velocity of the lateral mitral annulus with tissue Doppler imaging
SD: Standard deviation
TAPSE: Tricuspid annular plane systolic excursion
TTE: Transthoracic echocardiography
VTI: Velocity-time integral
