# Supplementary material for: What is the lowest change in cardiac output that transthoracic echocardiography can detect?
Source: Crit Care. 2019 Apr 11;23:116. doi: 10.1186/s13054-019-2413-x (PMC6458708; doi:10.1186/s13054-019-2413-x)
Supplement: Supplementary file 1 — Table S1. Patients’ characteristics at baseline. (DOCX 33 kb) [file 13054_2019_2413_MOESM1_ESM.docx]

**Table S1. Patients’ characteristics at baseline.**

| **Characteristics** | Whole population  (n=110) | Patients with atrial fibrillation  (n=18) | Patients with invasive mechanical ventilation  (n=59) |
| --- | --- | --- | --- |
|  |  |  |  |
| Age (years) | 66 ± 17 | 78 ± 12 | 65 ± 15 |
|  |  |  |  |
| SAPS II | 52 ± 19 | 55 ± 25 | 59 ± 19 |
|  |  |  |  |
| Body mass index (kg/m²) | 25 ± 5 | 25 ± 4 | 26 ± 5 |
|  |  |  |  |
| Gender (F/M) | 42 / 68 | 8/ 10 | 23 / 36 |
|  |  |  |  |
| Tidal volume (mL/kg of predicted body weight)* | 6.5 [5.9-7.8] | 7.6 [6.1-8.2] | 6.5 [5.9-7.8] |
|  |  |  |  |
| Respiratory rate (/min)* | 23 ± 5 | 25 ± 3 | 23 ± 5 |
|  |  |  |  |
| Plateau pressure (cm H_2_O)* | 20 ± 4 | 22 ± 4 | 20 ± 4 |
|  |  |  |  |
| PEEP (cm H_2_O)* | 8 ± 3 | 9 ± 2 | 8 ± 3 |
|  |  |  |  |
| Doses of norepinephrine (µg/kg/min) | 0.42 [0.18-0.71] | 0.40 [0.15-0.64] | 0.55 [0.25-0.97] |
|  |  |  |  |
| Lactate (mmol/L) | 1.3 [0.9-3.1] | 1.9 [0.9-3.5] | 1.8 [1.1-4.0] |
|  |  |  |  |

Data are summarised as mean ± standard deviation, median [interquartile range] or number.

*Concerning respiratory parameters, n=7 in patients with atrial fibrillation.

PEEP: positive end-expiratory pressure; SAPS: simplified acute physiology score.
