# Supplementary material for: What is the lowest change in cardiac output that transthoracic echocardiography can detect?
Source: Crit Care. 2019 Apr 11;23:116. doi: 10.1186/s13054-019-2413-x (PMC6458708; doi:10.1186/s13054-019-2413-x)
Supplement: Supplementary file 2 — Table S2. Haemodynamic parameters during the first and the third transthoracic echocardiography examinations. (DOCX 25 kb) [file 13054_2019_2413_MOESM2_ESM.docx]

**Table S2. Haemodynamic parameters during the first and the third transthoracic echocardiography examinations.**

| **Variables** | **First TTE examination** | |  | **Third TTE**  **examination** | |
| --- | --- | --- | --- | --- | --- |
|  |  |  |  |  |  |
| Heart rate (beats/min) | 91 | ± 19 |  | 91 | ± 19 |
|  |  |  |  |  |  |
| Systolic arterial pressure (mmHg) | 129 | ± 22 |  | 128 | ± 21 |
|  |  |  |  |  |  |
| Diastolic arterial pressure (mmHg) | 64 | ± 13 |  | 64 | ± 14 |
|  |  |  |  |  |  |
| Mean arterial pressure (mmHg) | 84 | ± 14 |  | 84 | ± 14 |
|  |  |  |  |  |  |
| Pulse contour analysis-derived CI (L/min/m²)* | 3.37 | ± 1.41 |  | 3.39 | ± 1.39 |
|  |  |  |  |  |  |

CI: cardiac index; TTE: transthoracic echocardiography.

n=100, data are summarised as mean ± standard deviation.

*n=20 for cardiac index measurement. †p<0.05 third *vs.* first TTE examinations.
