# Supplementary material for: What is the lowest change in cardiac output that transthoracic echocardiography can detect?
Source: Crit Care. 2019 Apr 11;23:116. doi: 10.1186/s13054-019-2413-x (PMC6458708; doi:10.1186/s13054-019-2413-x)
Supplement: Supplementary file 3 — Table S3. Intra-examination least significant change of transthoracic echocardiography measurements according to cardiac rhythm. (DOCX 28 kb) [file 13054_2019_2413_MOESM3_ESM.docx]

**Table S3. Intra-examination least significant change of transthoracic echocardiography measurements according to cardiac rhythm.**

|  | *One*  *measurement* | | *Two*  *measurements* | | | *Three*  *measurements* | | | *Four*  *measurements* | | | *Five*  *measurements* | | |
| --- | --- | --- | --- | --- | --- | --- | --- | --- | --- | --- | --- | --- | --- | --- |
| **TTE parameters** | *Atrial fibrillation (n=16)* | *Sinus*  *rhythm (n=84)* | | *Atrial fibrillation (n=16)* | *Sinus*  *rhythm (n=84)* | | *Atrial fibrillation (n=16)* | *Sinus*  *rhythm (n=84)* | | *Atrial fibrillation (n=16)* | *Sinus*  *rhythm (n=84)* | | *Atrial fibrillation (n=16)* | *Sinus*  *rhythm (n=84)* |
| **LV parameters** |  |  | |  |  | |  |  | |  |  | |  |  |
| E wave | 10 [6-19]% | 10 [5-15]% | | 7 [4-13]% | 7 [4-11]% | | 6 [4-11]% | 6 [3-9]% | | 5 [3-9]% | 5 [3-8]% | | 5 [3-8]% | 5 [2-7]% |
| A wave | - | 11 [5-15]% | | - | 8 [4-11]% | | - | 6 [3-9]% | | - | 6 [3-8]% | | - | 5 [2-7]% |
| e’ wave | 21 [10-44]% | 13 [7-20]%* | | 15 [7-31]% | 9 [5-14]%* | | 12 [6-26]% | 7 [4-11]%* | | 10 [5-22]% | 6 [3-10]%* | | 9 [5-20]% | 6 [3-9]%* |
| E/A ratio | - | 14 [7-20]% | | - | 10 [5-14]% | | - | 8 [4-12]% | | - | 7 [4-10]% | | - | 6 [3-9]% |
| E/e’ ratio | 26 [9-50]% | 17 [12-26]% | | 18 [6-35]% | 12 [8-18]% | | 15 [5-29]% | 10 [7-15]% | | 13 [5-25]% | 8 [6-13]% | | 12 [4-22]% | 8 [5-12]% |
| s’ wave | 17 [8-23]% | 12 [9-19]% | | 12 [6-16]% | 9 [6-13]% | | 10 [5-13]% | 7 [5-11]% | | 9 [4-12]% | 6 [4-9]% | | 8 [4-10]% | 5 [4-8]% |
| VTI | 20 [7-29]% | 9 [5-13]%* | | 14 [5-21]% | 6 [4-9]%* | | 12 [4-17]% | 5 [3-8]%* | | 10 [4-15]% | 4 [3-7]%* | | 9 [3-13]% | 4 [2-6]%* |
| LVEF | 24 [13-47]% | 19 [9-24]% | | 17 [10-33]% | 14 [7-17]% | | 14 [8-27]% | 11 [5-14]% | | 13 [7-24]% | 10 [5-12]% | | 11 [6-21]% | 9 [4-11]% |
|  |  |  | |  |  | |  |  | |  |  | |  |  |
| **RV parameters** |  |  | |  |  | |  |  | |  |  | |  |  |
| TAPSE | 22 [14-36]% | 11 [8-16]%* | | 15 [10-26]% | 8 [5-11]%* | | 13 [8-21]% | 6 [4-9]%* | | 11 [7-18]% | 5 [4-8]%* | | 10 [6-16]% | 5 [3-7]%* |
| S wave | 18 [12-26]% | 11 [6-17]%* | | 13 [9-18]% | 8 [4-12]%* | | 11 [7-15]% | 6 [3-10]%* | | 9 [6-13]% | 6 [3-8]%* | | 8 [5-11]% | 5 [3-7]%* |
|  |  |  | |  |  | |  |  | |  |  | |  |  |
| **LV and RV dimensions** |  |  | |  |  | |  |  | |  |  | |  |  |
| LVEDA | 7 [4-13]% | 10 [7-15]% | | 5 [3-9]% | 7 [5-11]% | | 4 [2-8]% | 6 [4-9]% | | 3 [2-7]% | 5 [3-8]% | | 3 [2-6]% | 4 [3-7]% |
| RVEDA | 15 [7-19]% | 13 [8-20]% | | 10 [5-13]% | 9 [6-14]% | | 9 [4-11]% | 8 [5-12]% | | 7 [4-9]% | 7 [4-10]% | | 7 [3-8]% | 6 [4-9]% |
| RVEDA/LVEDA | 15 [9-29]% | 16 [9-23]% | | 11 [6-20]% | 11 [7-16]% | | 9 [5-17]% | 9 [5-13]% | | 8 [4-15]% | 8 [5-12]% | | 7 [4-13]% | 7 [4-10]% |

n=100, data are summarised as median [interquartile range].*p <0.05 sinus rhythm *vs.* atrial fibrillation.

LV: left ventricular; RV: right ventricular; TTE: transthoracic echocardiography; E: early peak velocity of transmitral flow at pulsed Doppler; A: atrial peak velocity of transmitral flow at pulsed Doppler; e’: early diastolic peak velocity of the lateral mitral annulus at Tissue Doppler Imaging; s’: systolic peak velocity of the lateral mitral annulus at Tissue Doppler Imaging; VTI: velocity-time integral of the left ventricular outflow tract; LVEF: left ventricular ejection fraction; TAPSE: tricuspid annular plane systolic excursion; S: systolic peak velocity of the tricuspid annulus at Tissue Doppler Imaging; LVEDA: left ventricular end-diastolic area; RVEDA: right ventricular end-diastolic area.
