# Supplementary material for: What is the lowest change in cardiac output that transthoracic echocardiography can detect?
Source: Crit Care. 2019 Apr 11;23:116. doi: 10.1186/s13054-019-2413-x (PMC6458708; doi:10.1186/s13054-019-2413-x)
Supplement: Supplementary file 4 — Table S4. Intra-examination intra-observer variability of transthoracic echocardiography measurements according to cardiac rhythm and mechanical ventilation. (DOCX 26 kb) [file 13054_2019_2413_MOESM4_ESM.docx]

**Table S4. Intra-examination intra-observer variability of transthoracic echocardiography measurements according to cardiac rhythm and mechanical ventilation.**

| **TTE parameters** | *Atrial fibrillation (n=16)* | *Sinus rhythm*  *(n=84)* | *With invasive*  *mechanical ventilation (n=54)* | *Without invasive mechanical ventilation (n=46)* |
| --- | --- | --- | --- | --- |
| **LV parameters** |  |  |  |  |
| E wave | 5 [2-7]% | 3 [1-7]% | 3 [1-6]% | 5 [1-7]% |
| A wave^£^ | - | 4 [2-7]% | 3 [1-6]% | 5 [2-7]%† |
| e’ wave | 9 [6-18]% | 4 [1-9]%* | 5 [2-9]% | 6 [3-11]% |
| E/A ratio^£^ | - | 4 [2-10]% | 3 [2-8]% | 8 [3-12]%† |
| E/e’ ratio | 7 [5-20]% | 7 [3-12]% | 6 [2-12]% | 8 [4-15]% |
| s’ wave | 10 [4-15]% | 5 [3-8]% | 5 [3-8]% | 5 [3-12]% |
| VTI | 8 [3-16]% | 3 [2-7]%* | 4 [2-7]% | 4 [2-9]% |
| LVEF | 7 [4-18]% | 6 [2-13]% | 6 [2-13]% | 7 [2-13]% |
|  |  |  |  |  |
| **RV parameters** |  |  |  |  |
| TAPSE | 8 [5-15]% | 4 [0-7]%* | 4 [0-6]% | 6 [0-9]% |
| S wave | 7 [4-14]% | 4 [2-8]% | 4 [2-7]% | 5 [3-11]% |
|  |  |  |  |  |
| **LV and RV dimensions** |  |  |  |  |
| LVEDA | 4 [2-8]% | 5 [3-8]% | 5 [1-8]% | 4 [2-6]% |
| RVEDA | 5 [1-10]% | 5 [2-9]% | 4 [2-8]% | 6 [3-10]% |
| RVEDA/LVEDA ratio | 7 [3-11]% | 7 [4-11]% | 6 [3-11]% | 8 [4-11]% |

n=100. Data are summarised as median [interquartile range]. *p <0.05 sinus rythme *vs.* atrial fibrillation. †p <0.05 without *vs.* with invasive mechanical ventilation.

^£^Concerning the A wave and the E/A ratio, n=49 and n=35 in patients with and without invasive mechanical ventilation respectively.

LV: left ventricular; RV: right ventricular; TTE: transthoracic echocardiography; E: early peak velocity of transmitral flow at pulsed Doppler; A: atrial peak velocity of transmitral flow at pulsed Doppler; e’: early diastolic peak velocity of the lateral mitral annulus at Tissue Doppler Imaging; s’: systolic peak velocity of the lateral mitral annulus at Tissue Doppler Imaging; VTI: velocity-time integral of the left ventricular outflow tract; LVEF: left ventricular ejection fraction; TAPSE: tricuspid annular plane systolic excursion; S: systolic peak velocity of the tricuspid annulus at Tissue Doppler Imaging; LVEDA: left ventricular end-diastolic area; RVEDA: right ventricular end-diastolic area.
