# Supplementary material for: What is the lowest change in cardiac output that transthoracic echocardiography can detect?
Source: Crit Care. 2019 Apr 11;23:116. doi: 10.1186/s13054-019-2413-x (PMC6458708; doi:10.1186/s13054-019-2413-x)
Supplement: Supplementary file 5 — Table S5. Intra-examination precision of transthoracic echocardiography measurements in atrial fibrillation. (DOCX 26 kb) [file 13054_2019_2413_MOESM5_ESM.docx]

**Table S5. Intra-examination precision of transthoracic echocardiography measurements in atrial fibrillation.**

| **TTE parameters** | *One measurement* | *Two measurements* | *Three measurements* | *Four measurements* | *Five measurements* |
| --- | --- | --- | --- | --- | --- |
| **LV parameters** |  |  |  |  |  |
| E wave | 7 [4-13]% | 5 [3-9]% | 4 [3-7]% | 4 [2-7]% | 4 [2-6]% |
| A wave | - | - | - | - | - |
| e’ wave | 15 [7-31]% | 10 [5-22]% | 8 [4-18]% | 7 [4-16]% | 7 [3-14]% |
| E/A ratio | - | - | - | - | - |
| E/e’ ratio | 18 [6-35]% | 13 [5-25]% | 11 [4-21]% | 9 [3-18]% | 8 [3-16]% |
| s’ wave | 12 [6-16]% | 9 [4-12]% | 7 [4-9]% | 6 [3-8]% | 5 [3-8]% |
| VTI | 14 [5-21]% | 10 [4-15]% | 8 [3-12]% | 7 [3-11]% | 6 [2-9]% |
| LVEF | 17 [10-33]% | 13 [7-24]% | 10 [6-19]% | 9 [5-17]% | 8 [4-15]% |
|  |  |  |  |  |  |
| **RV parameters** |  |  |  |  |  |
| TAPSE | 15 [10-26]% | 11 [7-18]% | 9 [6-15]% | 8 [5-13]% | 7 [4-12]% |
| S wave | 13 [9-18]% | 9 [6-13]% | 8 [5-10]% | 7 [4-9]% | 6 [4-8]% |
|  |  |  |  |  |  |
| **LV and RV dimensions** |  |  |  |  |  |
| LVEDA | 5 [3-9]% | 3 [2-7]% | 3 [2-5]% | 2 [1-5]% | 2 [1-4]% |
| RVEDA | 10 [5-13]% | 7 [4-9]% | 6 [3-8]% | 5 [3-7]% | 5 [2-6]% |
| RVEDA/LVEDA | 11 [6-20]% | 8 [4-15]% | 6 [4-12]% | 6 [3-10]% | 5 [3-9]% |

n=16, data are summarised as median [interquartile range].

LV: left ventricular; RV: right ventricular; TTE: transthoracic echocardiography; E: early peak velocity of transmitral flow at pulsed Doppler; A: atrial peak velocity of transmitral flow at pulsed Doppler; e’: early diastolic peak velocity of the lateral mitral annulus at Tissue Doppler Imaging; s’: systolic peak velocity of the lateral mitral annulus at Tissue Doppler Imaging; VTI: velocity-time integral of the left ventricular outflow tract; LVEF: left ventricular ejection fraction; TAPSE: tricuspid annular plane systolic excursion; S: systolic peak velocity of the tricuspid annulus at Tissue Doppler Imaging; LVEDA: left ventricular end-diastolic area; RVEDA: right ventricular end-diastolic area
