# Supplementary material for: What is the lowest change in cardiac output that transthoracic echocardiography can detect?
Source: Crit Care. 2019 Apr 11;23:116. doi: 10.1186/s13054-019-2413-x (PMC6458708; doi:10.1186/s13054-019-2413-x)
Supplement: Supplementary file 7 — Table S7. Intra-examination least significant change of transthoracic echocardiography measurements according to mechanical ventilation. (DOCX 30 kb) [file 13054_2019_2413_MOESM7_ESM.docx]

**Table S7. Intra-examination least significant change of transthoracic echocardiography measurements according to mechanical ventilation.**

|  | *One*  *measurement* | | *Two*  *measurements* | | *Three*  *measurements* | | | *Four*  *measurements* | | | *Five*  *measurements* | | |
| --- | --- | --- | --- | --- | --- | --- | --- | --- | --- | --- | --- | --- | --- |
| **TTE parameters** | *With invasive mechanical ventilation (n=54)* | *Without invasive mechanical ventilation (n=46)* | *With invasive mechanical ventilation (n=54)* | *Without invasive mechanical ventilation (n=46)* | | *With invasive mechanical ventilation (n=54)* | *Without invasive mechanical ventilation (n=46)* | | *With invasive mechanical ventilation (n=54)* | *Without invasive mechanical ventilation (n=46)* | | *With invasive mechanical ventilation (n=54)* | *Without invasive mechanical ventilation (n=46)* |
| **LV parameters** |  |  |  |  | |  |  | |  |  | |  |  |
| E wave | 10 [5-16]% | 11 [7-16]% | 7 [3-11]% | 7 [5-11]% | | 6 [3-9]% | 6 [4-9]% | | 5 [2-8]% | 5 [3-8]% | | 4 [2-7]% | 5 [3-7]% |
| A wave^£^ | 9 [5-13]% | 14 [7-20]%* | 6 [4-9]% | 10 [5-14]%* | | 5 [3-7]% | 8 [4-12]%* | | 4 [3-6]% | 7 [3-10]%* | | 4 [2-6]% | 6 [3-9]%* |
| e’ wave | 12 [8-16]% | 18 [7-24]%* | 9 [6-12]% | 13 [5-17]%* | | 7 [5-9]% | 10 [4-14]%* | | 6 [4-8]% | 9 [4-12]%* | | 6 [4-7]% | 8 [3-11]%* |
| E/A ratio^£^ | 11 [6-19]% | 17 [13-27]%* | 8 [4-13]% | 12 [9-19]%* | | 6 [4-11]% | 10 [7-16]%* | | 6 [3-9]% | 8 [6-14]%* | | 5 [3-8]% | 7 [5-13]%* |
| E/e’ ratio | 17 [11-26]% | 18 [11-29]% | 12 [8-18]% | 13 [8-20]% | | 10 [6-15]% | 11 [6-16]% | | 8 [5-13]% | 9 [5-14]% | | 8 [5-11]% | 8 [5-13]% |
| s’ wave | 10 [7-19]% | 15 [9-19]% | 7 [5-13]% | 10 [6-13]% | | 6 [4-11]% | 8 [5-11]% | | 5 [3-9]% | 7 [5-9]% | | 5 [3-8]% | 7 [4-8]% |
| VTI | 8 [5-13]% | 11 [6-17]% | 6 [4-9]% | 8 [5-12]% | | 5 [3-7]% | 6 [4-10]% | | 4 [2-6]% | 5 [3-8]% | | 4 [2-6]% | 5 [3-8]% |
| LVEF | 19 [9-23]% | 20 [10-28]% | 14 [6-17]% | 14 [7-20]% | | 11 [5-13]% | 12 [6-16]% | | 10 [5-12]% | 10 [5-14]% | | 9 [4-10]% | 9 [5-12]% |
|  |  |  |  |  | |  |  | |  |  | |  |  |
| **RV parameters** |  |  |  |  | |  |  | |  |  | |  |  |
| TAPSE | 11 [8-18]% | 13 [8-20]% | 8 [6-13]% | 9 [6-14]% | | 7 [5-10]% | 7 [5-12]% | | 6 [4-9]% | 6 [4-10]% | | 5 [4-8]% | 6 [4-9]% |
| S wave | 12 [6-18]% | 12 [6-19]% | 8 [5-12]% | 9 [4-14]% | | 7 [4-10]% | 7 [4-11]% | | 6 [3-9]% | 6 [3-10]% | | 5 [3-8]% | 6 [3-9]% |
|  |  |  |  |  | |  |  | |  |  | |  |  |
| **LV and RV dimensions** |  |  |  |  | |  |  | |  |  | |  |  |
| LVEDA | 10 [7-15]% | 8 [6-13]% | 7 [5-11]% | 6 [4-9]% | | 6 [4-9]% | 5 [3-8]% | | 5 [3-8]% | 4 [3-7]% | | 5 [3-7]% | 4 [3-6]% |
| RVEDA | 12 [8-20]% | 15 [9-19]% | 8 [6-14]% | 10 [6-13]% | | 7 [5-13]% | 8 [5-11]% | | 6 [4-10]% | 7 [4-9]% | | 5 [4-9]% | 7 [4-8]% |
| RVEDA/LVEDA | 16 [9-24]% | 17 [9-23]% | 11 [6-17]% | 12 [6-16]% | | 9 [5-14]% | 10 [5-13]% | | 8 [4-12]% | 9 [4-12]% | | 7 [4-11]% | 8 [4-10]% |

n=100, data are summarised as median [interquartile range]. *p <0.05 without *vs.* with invasive mechanical ventilation.

^£^Concerning the A wave and the E/A ratio, n=49 and n=35 in patients with and without invasive mechanical ventilation respectively.

LV: left ventricular; RV: right ventricular; TTE: transthoracic echocardiography; E: early peak velocity of transmitral flow at pulsed Doppler; A: atrial peak velocity of transmitral flow at pulsed Doppler; e’: early diastolic peak velocity of the lateral mitral annulus at Tissue Doppler Imaging; s’: systolic peak velocity of the lateral mitral annulus at Tissue Doppler Imaging; VTI: velocity-time integral of the left ventricular outflow tract; LVEF: left ventricular ejection fraction; TAPSE: tricuspid annular plane systolic excursion; S: systolic peak velocity of the tricuspid annulus at Tissue Doppler Imaging; LVEDA: left ventricular end-diastolic area; RVEDA: right ventricular end-diastolic area.
