# Supplementary material for: What is the lowest change in cardiac output that transthoracic echocardiography can detect?
Source: Crit Care. 2019 Apr 11;23:116. doi: 10.1186/s13054-019-2413-x (PMC6458708; doi:10.1186/s13054-019-2413-x)
Supplement: Supplementary file 8 — Table S8. Precision, intra- and inter-observer variability of transthoracic echocardiography measurements between two examinations. (DOCX 31 kb) [file 13054_2019_2413_MOESM8_ESM.docx]

**Table S8. Precision, intra and inter-observer variability of transthoracic echocardiography measurements between two examinations.**

|  | **Same operator** | | **Different operator** | |
| --- | --- | --- | --- | --- |
|  | *Precision* | *Intra-observer variability* | *Precision* | *Inter-observer variability* |
| **LV parameters** |  |  |  |  |
| E wave | 6 [3-12]% | 4 [2-8]% | 8 [4-14]%* | 6 [3-10]%* |
| A wave^£^ | 6 [2-13]% | 4 [2-9]% | 9 [4-16]%* | 6 [3-12]%* |
| e’ wave | 12 [5-24]% | 8 [4-17]% | 14 [6-27]% | 10 [5-19]% |
| E/A ratio^£^ | 8 [4-14]% | 6 [3-10]% | 8 [3-17]% | 6 [2-12]% |
| E/e’ ratio | 17 [6-30]% | 12 [4-21]% | 15 [7-28]% | 11 [5-20]% |
| s’ wave | 10 [5-17]% | 7 [3-12]% | 13 [6-19]% | 9 [4-14]% |
| VTI | 8 [4-13]% | 6 [3-9]% | 10 [5-18]%* | 7 [4-13]%* |
| LVEF | 6 [3-11]% | 4 [2-7]% | 5 [2-13]% | 4 [2-9]% |
|  |  |  |  |  |
| **RV parameters** |  |  |  |  |
| TAPSE | 10 [4-19]% | 7 [3-14]% | 12 [5-24]% | 8 [4-17]% |
| S wave | 8 [4-17]% | 6 [3-12]% | 11 [5-22]%* | 8 [3-15]%* |
|  |  |  |  |  |
| **LV and RV dimensions** |  |  |  |  |
| LVEDA | 8 [5-15]% | 6 [4-10]% | 10 [5-19]% | 7 [4-13]% |
| RVEDA | 11 [5-22]% | 8 [3-16]% | 12 [7-23]% | 8 [5-16]% |
| RVEDA/LVEDA ratio | 12 [6-21]% | 8 [5-15]% | 12 [5-25]% | 9 [3-17]% |

**TTE parameters**

n=100, data are summarised as median [interquartile range]. *p<0.05 different *vs.* same operator.

^£^Concerning the A wave and the E/A ratio, n=84.

LV: left ventricular; RV: right ventricular; TTE: transthoracic echocardiography; E: early peak velocity of transmitral flow at pulsed Doppler; A: atrial peak velocity of transmitral flow at pulsed Doppler; e’: early diastolic peak velocity of the lateral mitral annulus at Tissue Doppler Imaging; s’: systolic peak velocity of the lateral mitral annulus at Tissue Doppler Imaging; VTI: velocity-time integral of the left ventricular outflow tract; LVEF: left ventricular ejection fraction; TAPSE: tricuspid annular plane systolic excursion; S: systolic peak velocity of the tricuspid annulus at Tissue Doppler Imaging; LVEDA: left ventricular end-diastolic area; RVEDA: right ventricular end-diastolic area.
