# Supplementary material for: What is the lowest change in cardiac output that transthoracic echocardiography can detect?
Source: Crit Care. 2019 Apr 11;23:116. doi: 10.1186/s13054-019-2413-x (PMC6458708; doi:10.1186/s13054-019-2413-x)
Supplement: Supplementary file 9 — Table S9. Variability of transthoracic echocardiography measurements between two examinations performed by the same operator according to cardiac rhythm. (DOCX 27 kb) [file 13054_2019_2413_MOESM9_ESM.docx]

**Table S9. Variability of transthoracic echocardiography measurements between two examinations performed by the same operator according to cardiac rhythm.**

|  | ***Precision*** | | ***Least significant change*** | | ***Intra-observer variability*** | |
| --- | --- | --- | --- | --- | --- | --- |
| **TTE parameters** | *Atrial fibrillation (n=16)* | *Sinus rhythm (n=84)* | *Atrial fibrillation (n=16)* | *Sinus rhythm (n=84)* | *Atrial fibrillation (n=16)* | *Sinus rhythm (n=84)* |
| **LV parameters** |  |  |  |  |  |  |
| E wave | 6 [3-14]% | 6 [3-12]% | 9 [5-19]% | 8 [4-17]% | 4 [2-9]% | 4 [2-8]% |
| A wave | - | 6 [2-13]% | - | 9 [3-18]% | - | 4 [2-9]% |
| e’ wave | 9 [4-21]% | 14 [5-25]% | 12 [6-29]% | 19 [7-36]% | 6 [3-15]% | 10 [4-18]% |
| E/A ratio | - | 8 [4-14]% | - | 11 [6-19]% | - | 6 [3-10]% |
| E/e’ ratio | 14 [5-28]% | 18 [6-30]% | 19 [7-40]% | 25 [9-41]% | 10 [4-20]% | 13 [4-21]% |
| s’ wave | 10 [7-14]% | 10 [4-17]% | 14 [9-20]% | 14 [6-24]% | 7 [5-10]% | 7 [3-12]% |
| VTI | 8 [4-12]% | 8 [4-13]% | 11 [6-17]% | 11 [5-18]% | 6 [3-9]% | 6 [3-9]% |
| LVEF | 8 [4-14]% | 6 [3-10]% | 12 [6-19]% | 8 [4-15]% | 6 [3-10]% | 4 [2-7]% |
|  |  |  |  |  |  |  |
| **RV parameters** |  |  |  |  |  |  |
| TAPSE | 6 [5-15]% | 10 [4-20]% | 8 [6-21]% | 14 [6-28]% | 4 [3-11]% | 7 [3-14]% |
| S wave | 8 [6-14]% | 8 [4-18]% | 12 [8-19]% | 12 [6-25]% | 6 [4-10]% | 6 [3-13]% |
|  |  |  |  |  |  |  |
| **LV and RV dimensions** |  |  |  |  |  |  |
| LVEDA | 8 [3-14]% | 8 [5-15]% | 12 [4-19]% | 12 [8-21]% | 6 [2-9]% | 6 [4-11]% |
| RVEDA | 7 [4-18]% | 12 [5-23]% | 9 [6-26]% | 17 [8-32]% | 5 [3-13]% | 8 [4-16]% |
| RVEDA/LVEDA ratio | 14 [7-25]% | 12 [6-21]% | 20 [10-35]% | 16 [9-30]% | 10 [5-17]% | 8 [4-15]% |

n=100, data are summarised as median [interquartile range].

LV: left ventricular; RV: right ventricular; TTE: transthoracic echocardiography; E: early peak velocity of transmitral flow at pulsed Doppler; A: atrial peak velocity of transmitral flow at pulsed Doppler; e’: early diastolic peak velocity of the lateral mitral annulus at Tissue Doppler Imaging; s’: systolic peak velocity of the lateral mitral annulus at Tissue Doppler Imaging; VTI: velocity-time integral of the left ventricular outflow tract; LVEF: left ventricular ejection fraction; TAPSE: tricuspid annular plane systolic excursion; S: systolic peak velocity of the tricuspid annulus at Tissue Doppler Imaging; LVEDA: left ventricular end-diastolic area; RVEDA: right ventricular end-diastolic area.
