# Supplementary material for: What is the lowest change in cardiac output that transthoracic echocardiography can detect?
Source: Crit Care. 2019 Apr 11;23:116. doi: 10.1186/s13054-019-2413-x (PMC6458708; doi:10.1186/s13054-019-2413-x)
Supplement: Supplementary file 10 — Table S10. Variability of transthoracic echocardiography measurements between two examinations performed by the same operator according to mechanical ventilation. (DOCX 27 kb) [file 13054_2019_2413_MOESM10_ESM.docx]

**Table S10. Variability of transthoracic echocardiography measurements between two examinations performed by the same operator according to mechanical ventilation.**

|  | ***Precision*** | | ***Least significant change*** | | ***Intra-observer variability*** | |
| --- | --- | --- | --- | --- | --- | --- |
| **TTE parameters** | *With*  *invasive mechanical ventilation (n=54)* | *Without*  *invasive mechanical ventilation*  *(n=46)* | *With*  *invasive mechanical ventilation (n=54)* | *Without*  *invasive mechanical ventilation*  *(n=46)* | *With*  *invasive mechanical ventilation (n=54)* | *Without*  *invasive mechanical ventilation*  *(n=46)* |
| **LV parameters** |  |  |  |  |  |  |
| E wave | 6 [2-12]% | 6 [3-12]% | 8 [2-17]% | 9 [4-17]% | 4 [1-8]% | 4 [2-8]% |
| A wave^£^ | 6 [3-13]% | 6 [2-12]% | 8 [4-18]% | 9 [3-17]% | 4 [2-9]% | 4 [1-9]% |
| e’ wave | 14 [5-27]% | 10 [5-22]% | 19 [6-38]% | 14 [7-30]% | 10 [3-19]% | 7 [4-15]% |
| E/A ratio^£^ | 7 [3-13]% | 9 [6-16]% | 7 [3-13]% | 9 [6-16]% | 5 [2-9]% | 6 [4-11]% |
| E/e’ ratio | 16 [6-30]% | 19 [5-28]% | 23 [9-42]% | 27 [7-40]% | 11 [4-21]% | 13 [3-20]% |
| s’ wave | 10 [5-17]% | 9 [4-15]% | 14 [8-24]% | 13 [5-22]% | 7 [4-12]% | 7 [3-11]% |
| VTI | 8 [4-13]% | 6 [3-11]% | 12 [5-18]% | 9 [5-16]% | 6 [3-9]% | 5 [2-8]% |
| LVEF | 6 [3-12]% | 6 [3-10]% | 9 [4-16]% | 8 [4-14]% | 4 [2-8]% | 4 [2-7]% |
|  |  |  |  |  |  |  |
| **RV parameters** |  |  |  |  |  |  |
| TAPSE | 10 [4-19]% | 11 [4-19]% | 14 [6-28]% | 15 [6-26]% | 7 [3-14]% | 8 [3-13]% |
| S wave | 8 [4-17]% | 8 [4-15]% | 12 [6-24]% | 11 [6-21]% | 6 [3-12]% | 5 [3-11]% |
|  |  |  |  |  |  |  |
| **LV and RV dimensions** |  |  |  |  |  |  |
| LVEDA | 8 [5-13]% | 11 [5-16]% | 11 [7-19]% | 15 [7-22]% | 5 [4-9]% | 8 [4-11]% |
| RVEDA | 13 [6-21]% | 10 [4-22]% | 19 [8-30]% | 14 [6-31]% | 9 [4-15]% | 7 [3-16]% |
| RVEDA/LVEDA ratio | 13 [6-21]% | 12 [7-20]% | 18 [9-30]% | 16 [9-29]% | 9 [4-15]% | 8 [5-14]% |

n=100, data are summarised as median [interquartile range].

^£^Concerning the A wave and the E/A ratio, n=49 and n=35 in patients with and without invasive mechanical ventilation respectively.

LV: left ventricular; RV: right ventricular; TTE: transthoracic echocardiography; E: early peak velocity of transmitral flow at pulsed Doppler; A: atrial peak velocity of transmitral flow at pulsed Doppler; e’: early diastolic peak velocity of the lateral mitral annulus at Tissue Doppler Imaging; s’: systolic peak velocity of the lateral mitral annulus at Tissue Doppler Imaging; VTI: velocity-time integral of the left ventricular outflow tract; LVEF: left ventricular ejection fraction; TAPSE: tricuspid annular plane systolic excursion; S: systolic peak velocity of the tricuspid annulus at Tissue Doppler Imaging; LVEDA: left ventricular end-diastolic area; RVEDA: right ventricular end-diastolic area.
